# Catalytic Degradation of Methyl Orange Using Fe/Ag/Zn Trimetallic Nanoparticles

**DOI:** 10.3390/nano16010060

**Published:** 2025-12-31

**Authors:** Masaku Kgatle, Keneiloe Khoabane, Ntsoaki Mphuthi, Gebhu Ndlovu, Nosipho Moloto

**Affiliations:** 1 DSTI/Mintek Nanotechnology Innovation Centre, Advanced Materials Division, Mintek, Private Bag X3015, Randburg 2125, South Africa; keneiloes@mintek.co.za (K.K.); ntsoakim@mintek.co.za (N.M.);; 2 Molecular Sciences Institute, School of Chemistry, University of the Witwatersrand, P/Bag 3, Johannesburg 2050, South Africa

**Keywords:** trimetallic nanoparticles, nanoscale zerovalent iron, Fe/Ag/Zn catalyst, methyl orange dye

## Abstract

The present study involves the synthesis of polyvinylpyrrolidone (PVP)-stabilized iron-based trimetallic nanoparticles with different metal addition sequences (Fe/Ag/Zn, Fe/Zn/Ag and Fe/(Zn/Ag)) using the sodium borohydride reduction method. In order to investigate the catalytic reactivity of the nanoparticles, a series of batch experiments were performed using methyl orange dye as a model pollutant. It was found that the Fe/Ag/Zn system showed the maximum catalytic activity compared to the other studied trimetallic systems. About 100% of the methyl orange dye was removed within 1 min and the second-order rate constant obtained was 0.0744 (mg/L)^−1^ min^−1^; the rate of reaction was higher than that of the other trimetallic systems. Furthermore, the effects of pH, initial dye concentration and nanoparticle dosage on the degradation of methyl orange were investigated. The results showed that the reactivity of the Fe/Ag/Zn trimetallic nanoparticles was highly dependent on the aforementioned parameters. Higher reactivity was obtained at lower pH, lower initial methyl orange dye concentration and higher nanoparticle dosage. Lastly, liquid chromatography–mass spectroscopy (LC-MS) was used to elucidate the reaction pathway and identify by-products from methyl orange degradation. The developed catalyst demonstrated exceptionally rapid and apparent degradation of methyl orange within one minute, outperforming previously reported bimetallic and trimetallic systems. This work reports a cost-effective nZVI-based trimetallic system containing minimal silver, which shows promising reactivity toward azo dye degradation and may be suitable for future application in textile wastewater treatment.

## 1. Introduction

The textile industry has been having an inordinate negative effect on the worldwide economic growth as it uses high quantities of water thereby leading to significant pressure on natural water resources [1]. Approximately 10,000 different types of dyes are used in the textile industry and 10–15% are lost in the dyeing processes [2]. Dye concentration values reported in textile industry effluents vary depending on the type of the industry, for instance, 10–800 mg/L [3,4,5]. Some of these dyes are difficult to remove from the aqueous phase due to their high solubility [2]. The effluent released from textile industries contains a mixture of dyes, metals and other pollutants [6,7]. Moreover, textile wastewater is usually categorized by high chemical oxygen demand (COD), biological oxygen demand (BOD), total organic carbon (TOC), color and salt content [8,9].

Dyes can generally be classified as either natural or synthetic [6]. Synthetic dyes are further categorized into different groups according to their chemical structures (such as azo, sulphur and anthraquinone types) and their application methods [10]. Among these, azo dyes containing the characteristic azo linkage (–N=N–) are the most extensively used in the textile industry. However, many azo dyes are carcinogenic and mutagenic, posing risks to human and aquatic life due to the ineffectualness of conventional water treatment plants and wastewater treatment strategies in eliminating the color and mutagenic properties of some dyes [11,12,13]. Furthermore, azo dye compounds are recalcitrant to biodegradation and persist in the environment due to their high stability, which is intended to meet consumer market demands regarding the longevity of colors in the fibers [14].

Traditional techniques for removing textile dyes from water include physical (e.g., sedimentation, flotation, filtration), chemical (e.g., ozonation, chlorination, ion exchange), and biological processes (e.g., aerobic and anaerobic digestion) [15]. However, these approaches have their own drawbacks and shortcomings. The biological methods are said to be time-consuming and have lower efficiencies compared to other methods [16] while physical treatments merely transfer pollutants between phases without achieving complete degradation [17]. Moreover, none of these methods fully mineralize the contaminants, frequently leading to secondary pollution [18]. To overcome these shortcomings, nanotechnology-based methods have been explored. Nanoparticles composed of transition metals such as Fe and Zn have been widely investigated for the treatment of various pollutants such as organic dyes [19,20], heavy metals [21], and chlorinated solvents [22]. Despite their advantages, monometallic nanoparticles often suffer from limited catalytic efficiency and stability. To address these drawbacks, researchers have developed multimetallic systems comprising two or more distinct metals to enhance reactivity and durability [23]. For example, Fe and Zn have been combined to form Fe/Zn bimetallic nanoparticles that efficiently degrade a variety of organic pollutants [16,24]. Similarly, nanoscale zerovalent iron (nZVI) has been coupled with other metals such as Ag, Pd, Ni or Pt to improve catalytic activity in wastewater treatment processes [25,26]. These bimetallic systems have been widely applied in the degradation of organic dyes in aqueous media, demonstrating enhanced degradation and adsorption efficiencies compared to their monometallic counterparts [24,25,27]. Wang and co-workers [27] reported that Fe/Cu bimetallic nanoparticles achieved markedly higher degradation rates for Orange II dye relative to nZVI alone. Extending this concept, multimetallic systems incorporating Fe and Ag have demonstrated enhanced catalytic activity due to improved electron transfer and redox cycling between the metallic components [28]. The introduction of Zn^0^ or Zn-based oxides into such systems can further augment performance by providing structural stability and facilitating charge transport. In our system, zinc oxide (ZnO) forms naturally as an oxidation product of Zn^0^ during synthesis and reaction, and ZnO-based materials are well-known for their redox versatility and defect-mediated catalytic behavior. Therefore, ZnO possesses favorable redox potentials and high chemical stability; as shown by [29], the creation of oxygen vacancies in ZnO promotes charge separation and electron mobility features that can synergistically strengthen redox cycling and overall catalytic efficiency when combined with Fe and Ag [29,30]. Gautam et al. [24] demonstrated that Fe/Zn nanoparticles effectively removed malachite green and Congo red dyes from aqueous solutions, with removal efficiency being strongly dependent on solution pH. Furthermore, Liang et al. [31] showed that incorporating Zn into Fe formed an amorphous Fe-Zn oxide which markedly enhanced visible light absorption, stabilized Fe^2+^ species, and accelerated Fe^3+^/Fe^2+^ redox cycling, resulting in significantly improved •OH generation and photo-Fenton degradation efficiency compared to pure Fe oxides.

Although bimetallic nanoparticles have proven to be effective, trimetallic systems represent an emerging research frontier. Unlike monometallic and bimetallic nanoparticles, trimetallic nanoparticles are composed of three different metals, offering unique properties due to the synergistic interactions among their constituent elements. Their unique metal combination enhances catalytic efficiency, conductivity, optical properties, and magnetism, while their core–shell structure improves stability, selectivity, and activity [32,33]. Previous studies have shown that trimetallic nanoparticles display superior catalytic performance compared to their bi- and monometallic analogs [32,34,35]. For instance, Basavegowda and co-workers [32] prepared Fe/Ag/Pt particles that displayed a better catalytic activity in the reduction of p-nitroaniline and decolorization of rhodamine-B when compared to the monometallic (Fe, Ag and Pt) and bimetallic (Fe/Ag and Fe/Pt) nanoparticles. Moreover, Khan [34] reported that Fe/Ag/Pd nanoparticles exhibited excellent catalytic dehydrogenation of formic acid. In our earlier work [36], we reported on Fe/Cu/Ag trimetallic nanoparticles which demonstrated significantly higher catalytic activity for methyl orange degradation than corresponding bimetallic systems.

In this study, nZVI-based trimetallic composite nanoparticles with low cost and high reactivity were developed. While Fe/Zn and Fe/Ag bimetallic nanoparticles have previously shown promising catalytic activity [24,26], Zn’s strong reducing nature can cause rapid oxidation, and Ag’s high cost limits large-scale applications. To address these challenges, this research introduces a novel approach by designing trimetallic nanoparticles with varying metal configurations (Fe/Zn/Ag 5:5:0.1, Fe/Ag/Zn 5:0.1:5, and Fe/(Zn/Ag) 5:(5:0.1)) incorporating minimal silver content. This work further delves into the parametric effects of the Fe/Ag/Zn system in the catalytic degradation of methyl orange dye in aqueous media, opening new doors for efficient and sustainable water treatment solutions. This innovative approach not only addresses the limitations of existing systems but also promises to revolutionize environmental remediation methods with cost-effective and high-performance nanomaterials.

## 2. Experimental Section

### 2.1. Reagents

The following chemicals were used for nanoparticle synthesis: iron sulphate heptahydrate (FeSO_4_·7H_2_O, ≥99.0%), sodium borohydride (NaBH_4_, ≥98.0%), polyvinylpyrollidone (PVP, MW 40 000), ethanol (99.9%), zinc acetate dihydrate (Zn(C_2_H_3_O_2_)_2_·2H_2_O), hydrochloric acid (HCl, 37.0%) and sodium hydroxide pellets (NaOH, ≥98.0%), all of which were all obtained from Sigma Aldrich, South Africa. The silver precursor, silver nitrate, (AgNO_3_, >99.5% purity) was supplied by Radchem laboratory supplies. Methyl orange dye was obtained from ACE Chemicals. N,N-Dimethylformamide used for the electrochemical studies was also sourced from Sigma Aldrich. All the chemicals and materials were used as received and deionized water was employed throughout the experimental procedures.

### 2.2. Nanoparticle Synthesis and Characterization

#### 2.2.1. Synthesis of Nanoparticles

**Scheme 1:**
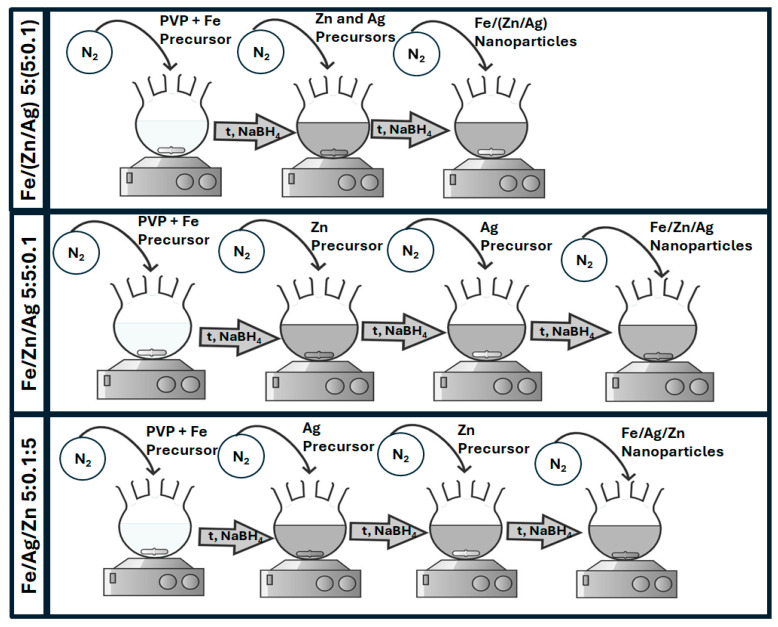
Summary of the nanoparticles’ synthesis procedure at different time (t) intervals (t = 30 min).

The composite trimetallic nanoparticles, prepared with varying metal addition sequences, were synthesized using the sodium borohydride reduction method. A typical synthesis involved adding 2 g of PVP, 50 mL of ethanol, and 50 mL of deionized water into a three-neck flask equipped with a magnetic stirrer. The solution was then degassed with nitrogen to remove oxygen and maintain an inert environment for nanoparticle formation. After 30 min, FeSO_4_·7H_2_O was added into the mixture and stirred for a further 30 min. A solution of NaBH_4_ was then added dropwise to the FeSO_4_·7H_2_O mixture under vigorous stirring, resulting in the formation of black nZVI nanoparticles. Subsequently, a solution of AgNO_3_, Zn(C_2_H_3_O_2_)_2_·2H_2_O, or both, depending on the desired trimetallic composition, was introduced into the nZVI suspension with continued stirring for 30 min. Then a solution of NaBH_4_ was added into the mixture in a dropwise manner. The reaction was stopped after 30 min for the Fe/(Zn/Ag) 5:(5:0.1) system. However, for Fe/Ag/Zn 5:0.1:5 and Fe/Zn/Ag 5:5:0.1 systems, after 30 min, Zn (C_2_H_3_O_2_)_2_·2H_2_O and AgNO_3_ were, respectively, added into the reaction vessel. Thereafter, a solution of NaBH_4_ was added into the mixture in a dropwise manner. Upon stirring for a further 30 min, the nanoparticles were removed from the stirrer and subsequently placed into centrifuge tubes. The nanoparticles were washed in ethanol and dried overnight under vacuum at 25 °C. The summary of the sequence of the addition of nanoparticle precursors is shown in Scheme 1.

#### 2.2.2. Characterization of Nanoparticles

Transmission electron microscopy (TEM) and energy dispersion X-ray spectroscopy (EDX) were performed on a JEOL JEM-2100F (JEOL, Tokyo, Japan) field emission electron microscope. The nanoparticles were suspended in ethanol by ultrasonication and subsequently drop-casted on a 300-mesh formvar-coated nickel grid from SPI supplies.

Scanning electron microscopy images were obtained using Zeiss Ultra 55 field emission microscope (FE-SEM, Carl Zeiss AG, Oberkochen, Germany). Images were captured using electron high tension of 2 kV, scan speed of 9 times per second and working distance of 2.3–2.4 mm.

The powder X-ray Diffraction (p-XRD) analysis of all samples was conducted on Bruker AXS D8 (Bruker, Johannesburg, South Africa) X-ray advanced powder diffractometer. The XRD was fitted with a LinxEye detector and nickel filter, using the Co Kα (1.78897 Å) wavelength operated at 40 kV and 35 mA. XRD patterns were recorded with a step size of 0.02 °/s in the 2θ range of 0 to 100° diffraction angle. In addition, XRD data was used to approximate the sizes of the particles using the Scherrer equation:

(1) $$D=\frac{K\lambda }{\beta Cos\theta }$$

where *D* is the size of the particle, *K* is the shape factor, *λ* is the wavelength of radiation expressed in nanometers, *θ* is the angle of diffraction of the peak in radians and *β* is the full width at half maximum of the peak expressed in radians [37].

The X-ray photoelectron spectroscopy (XPS) analysis of the trimetallic nanoparticles was conducted using a Thermo Fisher Scientific ESCAlab 250Xi (Thermo Scientific, Johannesburg, South Africa) with an Al kα light source at 1486.7 eV running at 300 W of power to identify individual elements in the nanoparticles. The hemispherical electron energy analyzer with a spot size of 900 µm worked in the constant analyzer energy (CAE) mode at an analyzer pass energy of 100 eV for the survey spectra and 20 eV for the high-resolution spectra of each element.

The specific surface area of the nanoparticles was determined using nitrogen adsorption–desorption measurements conducted on a Micromeritics ASAP 2020 surface area analyzer (Micromeritics Instrument Corporation, Norcross, GA, USA). Prior to analysis, the samples were degassed under vacuum at 100 °C for ~12 h to remove adsorbed moisture and volatiles. Nitrogen adsorption was carried out at −197.1 °C (77 K), and the specific surface area was calculated using the Brunauer–Emmett–Teller (BET) equation applied to the linear region of the adsorption isotherm (P/P_0_ = 0.05–0.30).

The surface charge of the trimetallic nanoparticles at different pH values was determined using a Malvern Zetasizer Nano ZS (Malvern Panalytical, Malvern, UK). Prior to measurement, 2 mg of each nanoparticle sample was dispersed in 10 mL of deionized water and ultrasonicated for 10 min to ensure homogeneous suspension. The pH of the suspension was adjusted to the desired value (pH 3–12) using dilute HCl or NaOH solutions. Each sample was equilibrated for 5 min and then transferred into a disposable folded capillary cell for analysis. Zeta potential values were recorded at 25 °C, and the average of three consecutive measurements was reported for each pH condition.

#### 2.2.3. Electrochemical Experiments

All electrochemical experiments were conducted using a computer-controlled Autolab Potentiostat/Galvanostat PGSTAT 302 N (Eco Chemie, Utrecht, The Netherlands), which was operated by NOVA 2.1.4 data processing software. Electrochemical impedance spectroscopy (EIS) measurements were performed with the integrated AUTOLAB frequency response analyzer (FRA32M). The data obtained from the EIS experiments were fitted using the Randles circuit model available in the NOVA software. The electrochemical properties of the nanoparticles were assessed using a standard three-electrode setup consisting of a 3 mm diameter modified glassy carbon electrode as the working electrode, a Ag/AgCl (3 M KCl) reference electrode, and a platinum wire counter electrode. To prepare the working electrodes, 5 mg of each catalyst was dispersed in 1 mL of DMF and sonicated for 10 min. A 10 μL sample of the resulting suspension was then drop-cast onto the glassy carbon surface and allowed to dry at room temperature for 30 min. The same mass loading and deposition protocol was employed for all electrodes to ensure reproducible and comparable catalyst films. Cyclic voltammetry (CV), linear sweep voltammetry (LSV), and electrochemical impedance spectroscopy (EIS) were used to evaluate the electrochemical behavior of the modified electrodes. CV measurements were carried out over a potential window of −1.5 to 0.0 V at a scan rate of 0.05 V/s, while Tafel plots were derived from LSV data recorded between −1.5 and −1.0 V at a scan rate of 0.02 V/s. EIS spectra were collected over the frequency range of 100 kHz to 0.1 Hz using a 0.05 Vrms sinusoidal modulation. All potentials are quoted versus Ag/AgCl. All electrochemical tests were conducted in 0.5 M Na_2_SO_4_ supporting electrolyte (pH 6), in the presence and in the absence of 10 ppm of methyl orange.

### 2.3. Methyl Orange Degradation Tests and Analysis

Batch experiments of methyl orange (MO) dye degradation were carried out to measure the catalytic activity of the synthesized nanoparticles. All experiments were conducted in an open-batch system under ambient laboratory light (natural light) conditions at room temperature. About 50 mL of 10 mg/L MO dye solution was placed in a beaker equipped with a magnetic stirrer bar and stirred on a magnetic stirrer plate at 250 rpm for 30 min. This was followed by addition of 10 mg of the nanoparticles and continued stirring at 250 rpm. Aliquots were drawn into small centrifuge tubes at the following time intervals: 1, 2, 3, 4, 5, 10, 15, 20 and 30 min. These were then quickly centrifuged for 10 min at 12,000 rpm to separate the nanoparticles from the liquid. Parametric tests were conducted by varying the pH, nanoparticle dosage and initial MO concentration. The pH of the solution was varied from 3 to 12 using HCl and NaOH to adjust, the nanoparticle dosage was varied from 4 to 10 mg and the initial MO concentration ranged from 10 to 300 mg/L. The tests were performed under the same experimental conditions as above. The samples were analyzed using a Thermo Fisher Scientific, Multiskan GO, UV-vis Microplate Spectrophotometer (Thermo Fisher Scientific, Vantaa, Finland). The degradation efficiency was calculated using the following equation:

(2) $$Degradation\left(\%\right)=\frac{A_{0}-\;A_{t}}{A_{0}}\;\times 100$$

where *A*_0_ is the MO dye absorbance before degradation and *A_t_* is the MO dye absorbance at particular time intervals *t*. The reactivity of surface sites of each of the trimetallic nanoparticles was determined using turnover number (*TON*) and turnover frequency (*TOF*). The *TON* was calculated using the number of active sites as follows:

(3) $$TON=\frac{\left(DE\%)(moles\;of\;dye\right)}{Number\;of\;active\;sites\;(moles)}$$

where *DE* is the dye degradation efficiency. *TOF*, which is the number of MO dye molecules that one catalytic site can convert into a product [38], was calculated at 5 min reaction time using the following equation:

(4) $$TOF=\frac{TON}{time}$$

### 2.4. Kinetic Study

The kinetic study of the degradation of MO was conducted using the zeroth-, first- and second-order kinetic models. The following differential rate equations were used to define the zeroth-, first- and second-order kinetic models, respectively:

(5) $$-\frac{dC_{t}}{dt}=k_{0}$$

(6) $$-\frac{dC_{t}}{dt}=k_{1}C_{t}$$

(7) $$-\frac{-dC_{t}}{dt}=k_{2}(C_{t}{)}^{2}$$

where *t* is the reaction time and *C_t_* is the dye concentration at time, *t*. Moreover, *k*_0_, *k*_1_ and *k*_2_ are the apparent rate constants of the zeroth-, first- and the second-order kinetic models. The integration of Equations (5)–(7) yields the following Equations (8)–(10), respectively:

(8) $$C_{t}=C_{0}-k_{0}t$$

(9) $$C_{t}=C_{0}e^{-k_{1}t}$$

(10) $$\frac{1}{C_{t}}=\frac{1}{C_{0}}+k_{2}t$$

where *C*_0_ is the initial dye concentration. The linear equation of the first-order kinetic model integrated Equation (9) is as follows:

(11) $$lnC_{t}=lnC_{0}-k_{1}t$$

The rate constants for all the kinetic models were determined from the slope of their linear plots.

### 2.5. Analysis of Methyl Orange Degradation Products and Pathway

The analysis of MO dye and its degradation products was conducted on a Bruker Compact Q-TOF high-resolution Liquid Chromatography-Mass Spectrophotometer (Bruker, Johannesburg, South Africa). Separations were achieved utilizing a Luna Omega 1.6 µm C18 column (50 × 2.1 mm) (Separations Scientific SA (Pty) Ltd., Johannesburg, South Africa) and a mobile phase composed 0.1% formic acid, in both acetonitrile and water under isocratic conditions, with a flow rate of 0.3 mL/min. A total of 20 µL of sample was injected using the autosample system. The MS was coupled with an electrospray ionization source (ESI) and operated at positive polarity. The ESI conditions were as follows: capillary voltage = 4500 V, endplate offset = –500 V, nebuliser pressure = 1.8 bar, drying gas flow = 9.0 L/min, temperature = 220 °C and the mass range was 50–1300 m/z.

## 3. Results and Discussion

### 3.1. Characterization of the Catalyst

**Figure 1:**
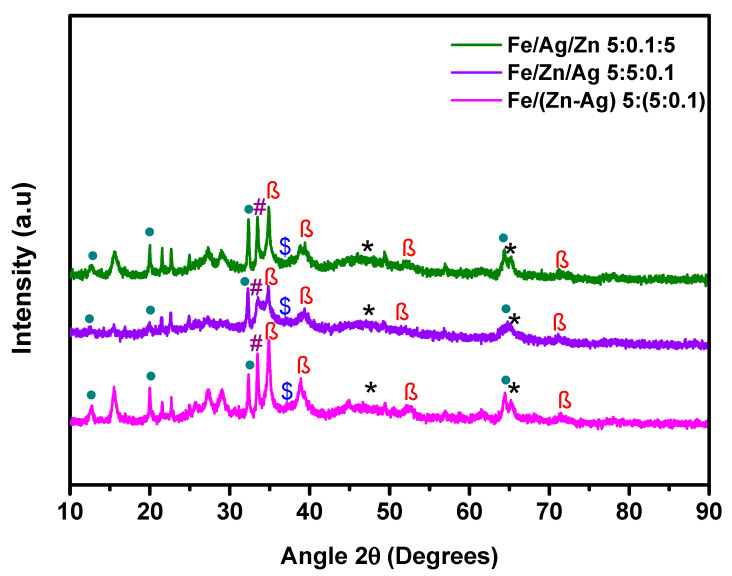
X-ray diffraction (XRD) patterns of the trimetallic nanoparticles and standard XRD plots of Fe, Zn, ZnO and Fe_2_O_3_. (Peaks of Fe^0^: *, peaks of Fe_2_O_3_: ●, peaks of Ag: $, peaks of Zn: β and peaks of ZnO: #).

The XRD results of synthesized trimetallic nanoparticles (Fe/Zn/Ag, Fe/(Zn/Ag) and Fe/Ag/Zn) are presented in Figure 1. The XRD patterns of the trimetallic nanoparticles depicted the presence of peaks at the 2θ of 45.0° and 66.0° corresponding to nZVI indexed at (110) and (200) crystalline planes, respectively. Iron oxide (Fe_2_O_3_ and Fe_3_O_4_) peaks appear at 2θ values of 12.0°, 20.0°, 22.5°, 24.9°, 26.0°, 30.3°, 34.0° and 62.5° [39,40]. The presence of these iron oxide peaks in the nanoparticles provide evidence of the nZVI layered structure, which comprises a body-centered cubic ɑ-Fe core covered by an oxide shell [39,41]. The existence of oxide peaks in nZVI-based nanoparticles is not a foreign phenomenon as it was also observed in previous studies [26,35,42,43,44]. The iron oxide might have been formed during the process of nanoparticle synthesis, nanoparticle drying or storing before the nanoparticles could be analyzed [26,42]. Furthermore, Zhou et al. [43] presented evidence of increasing iron oxide intensity in the bimetallic Fe/Pd nanoparticles as they age. However, Yuan et al. [35] showed that although the nanoparticles had moderately oxidized, the prepared Fe/Cu/Ag trimetallic nanoparticles were still highly effective in the degradation of p-nitrophenol and the corrosion products did not accrue on the surface of the trimetallic nanoparticles. This demonstrated that the presence of Cu and Ag on the surface of nZVI could impede its passivation and increase its lifespan.

The Fe^0^ peak intensity at 2θ of 45.0° in all trimetallic nanoparticles spectra decreases and widens as compared with the standard Fe XRD pattern. According to studies, the broadening of peaks is due to the reduction in the particle size and stabilization of the nanomaterial [45,46]. A smaller particle size of the crystals increases the number of defects in the crystalline network due to the reduced number of atoms available for crystallite assembly, leading to diffraction peaks with lower intensity and widening [45]. Moreover, the small peak at the 2θ value of 37.5° corresponding to the (111) crystalline plane indicates the presence of pure Ag [47]. The peaks at 2θ values of 34.4°, 39.6°, 53.5° and 71.9° correspond to pure hexagonal Zn. The ZnO peak can be observed at 2θ value of 33.5° [48,49]. Like Fe, Zn (E° = −0.76 V) is also prone to oxidation but has a lower reduction potential than Fe (E° = −0.447 V) [50], which makes Zn more reactive than Fe [22]. Gautam et al. [24] prepared Fe/Zn bimetallic nanoparticles and the XRD results showed the presence of Fe and ZnO. The findings reveal that alongside oxide phases, Fe, Zn, and Ag coexist within the trimetallic nanoparticles while maintaining their individual crystalline structures. All three samples display similar crystalline features, although the Ag peaks are weak or nearly invisible owing to the small amount of silver present. The obtained XRD results confirm that the nanoparticles possess a multiphase composite structure, comprising nZVI cores with oxide shells and dispersed Zn/ZnO and trace Ag domains, consistent with a core–shell multiphase trimetallic composite rather than a homogeneous alloy. The crystallite sizes determined by the Scherrer equation using all high intensity XRD peaks of the Fe/Ag/Zn, Fe/Zn/Ag and Fe/(Zn/Ag) nanoparticles were 18.9, 21.5 and 23.2 nm, respectively.

**Figure 2:**
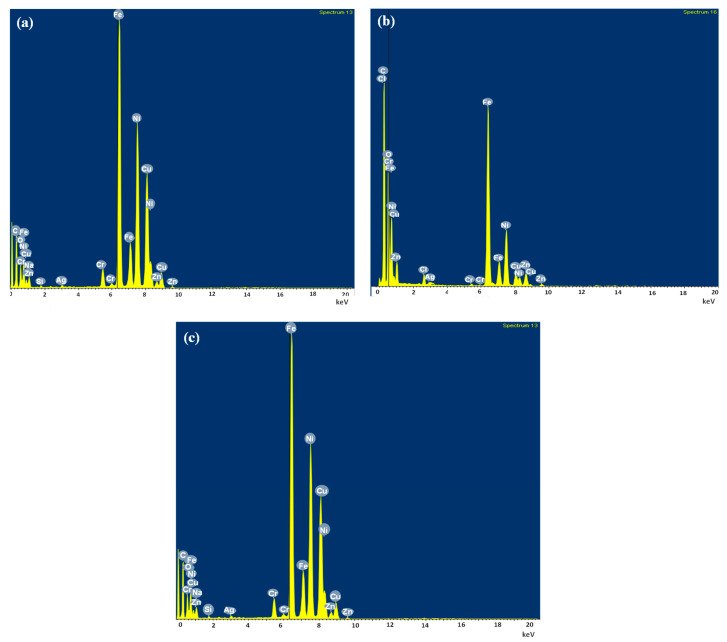
Energy-dispersive X-ray analysis of the trimetallic nanoparticles: (**a**) Fe/(Zn/Ag) 5(5:0.1), (**b**) Fe/Zn/Ag 5:5:0.1 and (**c**) Fe/Ag/Zn 5:0.1:5.

Figure 2 shows the EDX spectra of the synthesized trimetallic nanoparticles, which confirms their elemental composition. The EDX spectra show the presence of the main elements Fe at 0.7, 6.4 and 7.2 keV, Ag at 3.0 keV, Zn at 8.6 and 9.5 keV and O at 6 keV, as also observed in the XRD patterns. The presence of O in the spectra indicates that there were oxides co-existing with the trimetallic nanoparticles due to the ease of oxidation of both Fe and Zn [41]. The presence of Ni is attributed to the grid, C is from the PVP used to stabilize the nanoparticles while the other elements could simply be impurities emanating from various sources including the EDX detector [51]. The minor Cr and Cl signals observed are attributed to trace impurities originating from the Fe and Zn precursors, as well as possible contributions from the sample holder and residual salts used during synthesis.

**Figure 3:**
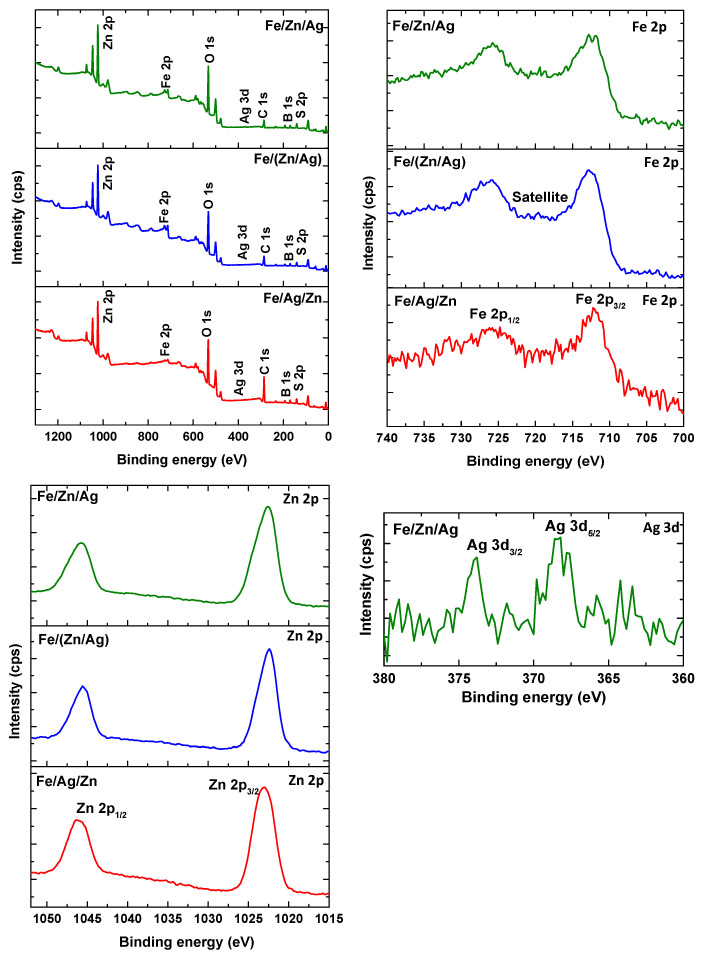
Survey spectra of the trimetallic nanoparticles: Fe/(Zn/Ag) 5(5:0.1), Fe/Zn/Ag 5:5:0.1 and Fe/Ag/Zn 5:0.1:5, their Fe 2p and Zn 2p high-resolution spectra and Ag 3d high-resolution spectrum of Fe/Zn/Ag 5:5:0.1 nanoparticles.

A further analysis of the trimetallic nanoparticles was carried out using XPS technique, which is a surface sensitive technique used for elemental identification within the surface of a material. The wide-scan survey and high-resolution spectral analyses were conducted; the high-resolution scan was used for the detailed chemical analysis of the elements identified in the full survey spectra. In Figure 3, the survey spectra of all the trimetallic nanoparticles show the presence of Fe, Zn, O and C; however, the XPS analysis of the Fe/Zn/Ag trimetallic system also detected the presence of Ag. Ag was not observed in the Fe/Ag/Zn and Fe/(Zn/Ag) systems because it is present in small amounts, whilst the other two metals are present in large quantities, likely obscuring the Ag. However, even though Ag was not detected using XPS in the aforementioned trimetallic systems, it was detected using XRD and EDX techniques. The C and O emanate from PVP and oxidation of Zn and Fe, respectively. These results corroborate those obtained with EDX and XRD analyses. The atomic percentages of each element present in the nanoparticles are summarized in Table 1. All three trimetallic nanoparticles show a high atomic percentage of O and C which have been attributed to the presence of iron and zinc oxides and the PVP used as the surfactant, respectively. Despite the same amounts being employed in the ratios, the atomic percentage of Zn is significantly higher than that of Fe in all three systems. The amount of Ag in the Fe/Zn/Ag system is much lower than the amounts of Fe and Zn, which is compatible with the ratio used.

The Fe 2p high-resolution spectra for all three trimetallic nanoparticles have two prominent photoelectron peaks at ~725 eV and ~712 eV which represent the binding energies of Fe 2p_1/2_ and Fe 2p_3/2_, respectively. The presence of ZVI is indicated by the aforementioned peaks, as well as a smaller band at a binding energy of ~706 eV [52]. The small peak at ~720 eV is a shake-up satellite which confirms the oxidation of Fe. The presence of a fraction of iron oxides and nZVI demonstrates that the nanoparticles have a core shell structure [52].

The XPS narrow spectra of Zn 2p shows two peaks at binding energies of ~1045 eV and ~1022 eV corresponding to the Zn 2p_1/2_ and Zn 2p_3/2_ of Zn^2+^, respectively [53]. The difference in binding energy between these two peaks is about 23 eV, which is consistent with the zinc metal’s published values [54]. Silver metal was detected only in the Fe/Zn/Ag trimetallic system and the Ag 3d XPS narrow spectrum is also displayed in Figure 3. The presence of pure silver is indicated by peaks at binding energies of 368.4 eV and 374.0 eV corresponding to Ag 3d_5/2_ and Ag 3d_3/2_, respectively. Furthermore, the peak at binding energy of 190 eV corresponding to B 1s likely originates from surface contamination or residual boron species from the NaBH_4_ reducing agent used during synthesis.

**Figure 4:**
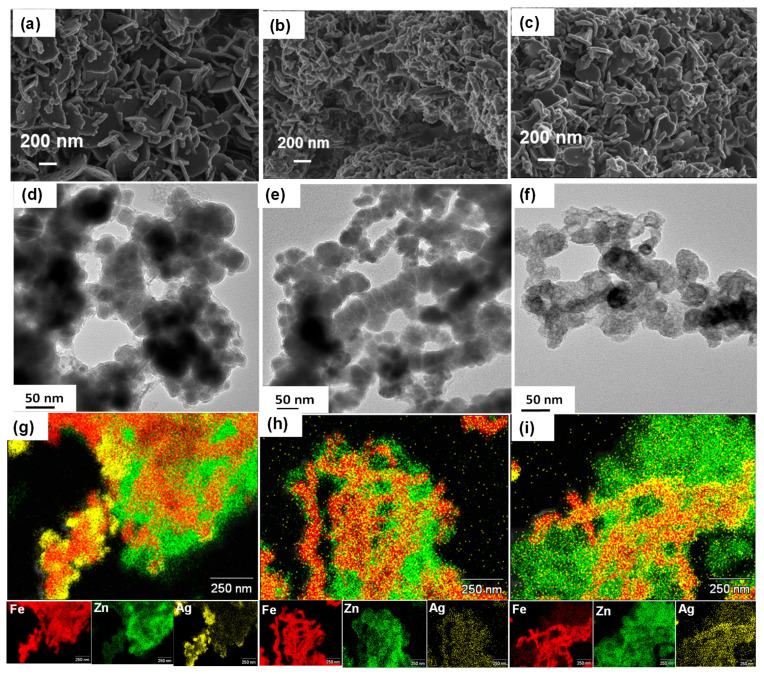
SEM images of (**a**) Fe/(Zn/Ag) 5:(5:0.1), (**b**) Fe/Ag/Zn 5:0.1:5 and (**c**) Fe/Zn/Ag 5:5:0.1, TEM images of (**d**) Fe/(Zn/Ag) 5:(5:0.1), (**e**) Fe/Ag/Zn 5:0.1:5 and (**f**) Fe/Zn/Ag 5:5:0.1, and elemental maps of (**g**) Fe/(Zn/Ag) 5:(5:0.1), (**h**) Fe/Ag/Zn 5:0.1:5 and (**i**) Fe/Zn/Ag 5:5:0.1.

The surface morphological properties of the products were conducted through the use of SEM, and the resulting images are displayed in Figure 4a–c for Fe/(Zn/Ag), Fe/Ag/Zn and Fe/Zn/Ag, respectively. An assembly of well-defined 2D plate-like trimetallic nanoparticles are observed for Fe/(Zn/Ag) and Fe/Zn/Ag. The well-defined plate-like form was not maintained for the Fe/Ag/Zn trimetallic nanoparticles as an assembly of mixed morphologies consisting of predominantly plate-like and lesser botryoidal texture was observed. The disparity in morphological structures between Fe/Ag/Zn and other trimetallic nanoparticles is attributed to the particle agglomeration and smaller particle size. The structures and sizes of the nanostructures rely heavily on the synthesis method and experimental conditions. In this case, it is clearly observed that the different metal addition sequences (Fe/Ag/Zn, Fe/Zn/Ag and Fe/(Zn/Ag) influence the morphological properties of the nanostructures.

Furthermore, transmission electron microscopy (TEM) was used for the characterization of the nanoparticles in order to achieve high accuracy of the real particle size and shape [55]. The TEM images for the trimetallic nanoparticles are presented in Figure 4d–f. The complex structures of trimetallic nanoparticles constitute a mixture of core–shell structures and multiphase trimetallic composite structures [56,57]. The images show a morphology that is quasispherical and particles arranged in chain-like aggregates due to the magnetic dipole–dipole interactions between the particles and thus creating an arrangement of magnetic clusters [45]. This kind of structure has been reported in most studies involving nZVI and nZVI-based bimetallic nanoparticles [20,43,58,59]. Moreover, the trimetallic nanoparticles in Figure 4d,e show a core–shell formation that reflects the oxidized part that surrounds the nZVI together with the other metals (Ag and Zn) [60]. The core–shell structure of nZVI nanoparticles has been reported before with the use of the NaBH_4_ chemical reduction method for nanoparticle synthesis [61,62]. Furthermore, the core–shell structure is produced as a result of successive reduction in the nanoparticles involving the deposition of a secondary and/or tertiary metal on the surface of nZVI [57]. The pre-synthesized nZVI is chemically surrounded by the deposited metal/s [57], in this case, both the metals and the oxides. The TEM results are congruous with the XRD results, revealing the crystalline structures of the nanoparticles notwithstanding the agglomeration. The Fe/Zn/Ag (Figure 4f) image has more irregularly shaped agglomerates that differ from the other images due to the formation of a more multiphase trimetallic composite. The Zn or ZnO nanoparticles are supposedly attached to the ends of polymer chains with micelles on the inside [63]. Thus, in addition, the irregular agglomerates could be due to the formation of unique polymer–surfactant complexes and free micelles [63]. It can also be observed from the XRD pattern of the Fe/Zn/Ag trimetallic system that some of the peaks are not as sharp as the other trimetallic nanoparticles’ peaks showing a more amorphous structure [64]. This, therefore, shows that the crystalline structure of the Fe/Zn/Ag trimetallic system is not as good as those of the other trimetallic systems [65,66].

Moreover, to verify the existence and distribution of Fe, Zn and Ag on the three samples, the EDS elemental maps were acquired and are shown in Figure 4g–i for Fe/(Zn/Ag), Fe/Ag/Zn and Fe/Zn/Ag, respectively. The elemental maps revealed the distribution and presence of Fe, Zn and Ag, elements, respectively. In all EDS mappings, the distinctive chain-like structure of the Fe is prominently evident, contrasting with the uneven distribution of the Zn and Ag in the images. However, in Figure 4h, depicting the Fe/Ag/Zn nanoparticles, a more pronounced morphology is evident, and the distribution of Zn and Ag on the surface of nZVI exhibits a greater degree of uniformity.

Particle sizes of the nanoparticles were determined using image J software (version 1.51, National Institute of Health, Bethesda, MD, USA). The particle size histograms of all the nanoparticles are shown in Supplementary Figure S2. The estimated average particle sizes of the Fe/Ag/Zn, Fe/Zn/Ag and Fe/(Zn/Ag) are 37, 35 and 45 nm, respectively. These are higher than the sizes obtained by XRD. This could be because XRD generally determines the crystallite size of which one particle can have many crystallites [67]; its more advantageous in cases where the particles are highly agglomerated [68]. These results are consistent with what other researchers have reported when comparing TEM and XRD particle sizes where the XRD size is normally smaller or equal to the TEM size [69]. Further quantitative techniques such as ICP-MS or CO-probe adsorption could provide more precise information on surface site density and will be pursued in future studies.

### 3.2. Effect of Metal Addition Sequence on Methyl Orange Degradation

**Figure 5:**
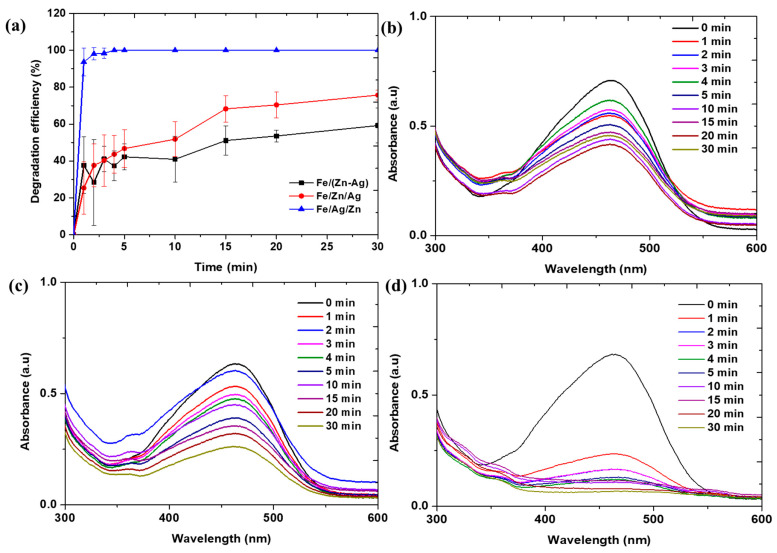
Degradation efficiencies of (**a**) 10 mg/L methyl orange dye using 10 mg of the trimetallic nanoparticles (Fe/Zn/Ag 5:5:0.1, Fe/Ag/Zn 5:0.1:5 and Fe/(Zn/Ag) 5:(5:0.1)) and UV-Vis absorption spectra of (**b**) Fe/(Zn/Ag) 5:(5:0.1), (**c**) Fe/Zn/Ag 5:5:0.1, and (**d**) Fe/Ag/Zn 5:0.1:5 trimetallic nanoparticles.

The results of the degradation profiles of MO and corresponding UV-Vis absorption profiles of the three trimetallic nanoparticles are presented in Figure 5. The figure presents the rapid degradation of MO (pH ~4.5) using the Fe/Ag/Zn as compared to the slow degradation using the other trimetallic systems. It can be noted that the Fe/Ag/Zn nanoparticles removed 100% of MO dye within 5 min into the reaction. This great reactivity of the Fe/Ag/Zn trimetallic nanoparticles could be a result of their smaller crystallite size compared to the other trimetallic systems. It has been established that smaller crystallite/nanoparticle sizes lead to an enhanced reactivity [70,71]. Bransfield et al. [72] and Yuan et al. [35] have demonstrated that the reactivity of the trimetallic system increases if the metals added onto the surface of iron have an increasing standard reduction potential (E°) trend. For example, in the trimetallic system Fe/Cu/Ag prepared by Yuan et al. [35] the order of metal plating was as follows: Ag/Ag^+^ (E° = 0.7996 V) > Cu/Cu^2+^ (E° = 0.3419 V) > Fe/Fe^2+^ (E°= −0.447 V). Thus, in this case, the high activity of Fe/Ag/Zn could be attributed to the fact that both Fe and Zn are strong reducing agents and are capable of releasing electrons that are required to generate atomic hydrogen that facilitates the reaction in the presence of water or dissolved oxygen [73,74]. Moreover, the incorporation of Ag nanoparticles enhances interfacial charge transfer and modifies the electronic structure of the composite, facilitating more efficient electron mobility and suppressing charge recombination [28,75]. This improved charge separation accelerates redox reactions and contributes to higher catalytic efficiency [28].

In addition, the sequence of metallic Zn^0^ and Ag^0^ deposition onto Fe^0^ plays a critical role in determining the final surface chemistry and reactivity of the trimetallic systems. In the Fe/Ag/Zn system, Ag^0^ is first deposited on Fe^0^ via galvanic displacement, followed by Zn^0^ deposition, which promotes more uniform surface coverage and improved interfacial contact among Fe, Ag, and Zn-derived oxide phases formed during reaction. In contrast, when Zn^0^ is introduced prior to Ag^0^, as in Fe/Zn/Ag and Fe/(Zn/Ag), competitive surface coverage and partial passivation of Fe^0^ can occur, limiting access to highly reactive Fe^0^ sites. These differences in metallic deposition order directly contribute to the observed variations in electron transfer efficiency and degradation performance among the three trimetallic systems.

The bimetallic Fe/Zn and Fe/Ag systems were also studied, and the results are presented in Figure S3. These bimetallic systems show an overall good performance as compared to the Fe/Zn/Ag and Fe/(Zn/Ag) trimetallic systems. However, the Fe/Ag/Zn system still had the highest degradation efficiency as compared to the bimetallic systems. Moreover, in a trimetallic system, the reactivity is thought to be more influenced by the second metal on the surface of iron (Zn in the Fe/Ag/Zn system) than the first one (Ag) [76]. This is despite the exceptions of either the obscuring of the first metal deposited onto nZVI by the second metal or by the deposition of the second metal on some non-accessible reactive sites by MO at the surface of nZVI particles [76].

**Figure 6:**
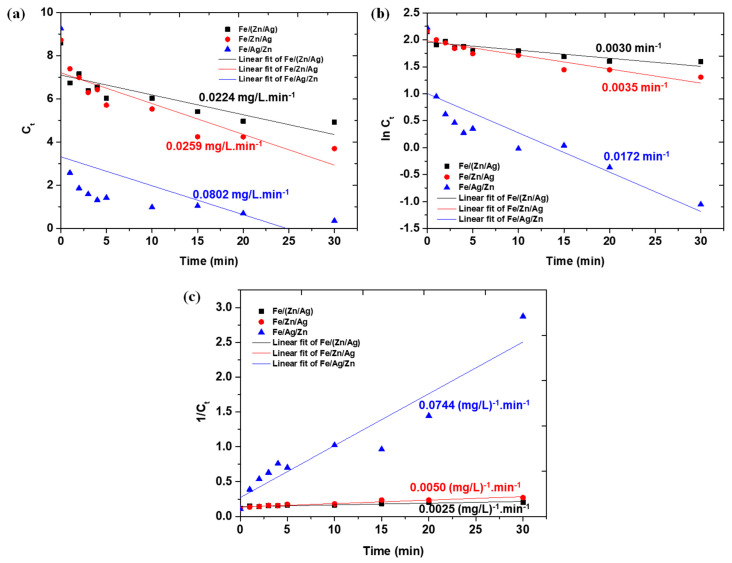
Kinetic plots of the synthesized nanoparticles: (**a**) zeroth-order, (**b**) first-order and (**c**) second-order with their corresponding k-values.

Both adsorption and degradation kinetics were evaluated during the optimization stage across pH, initial dye concentration, and catalyst loading. Adsorption was found to be negligible relative to catalytic degradation and did not significantly influence the overall removal behavior; therefore, the kinetic analysis in this section focuses exclusively on degradation processes. The degradation kinetics of MO using the trimetallic nanoparticles were fitted to three kinetic models, the zeroth-, first- and second-order models, and the plots are presented in Figure 6. As observed, the slope of the Fe/Ag/Zn plot in all kinetic models steepened, thereby signifying that the k_0_, k_1_ and k_2_ of the zeroth-, first- and second-order models, respectively, were higher than those of the other trimetallic systems. The R^2^ values of the three kinetic models are summarized in Table 2. It can be observed from the table that the second-order kinetic model best described the degradation of MO dye and the R^2^ values as above 0.80. Thus, the second-order rate constants for the reaction of the nanoparticles with MO were as follows: Fe/Ag/Zn (0.0744 (mg/L)^−1^ min^−1^) > Fe/Zn/Ag (0.0050 (mg/L)^−1^ min^−1^) > Fe/(Zn/Ag) (0.0025 (mg/L)^−1^ min^−1^). The reactivity of the Fe/Ag/Zn trimetallic system was found to be 15 times higher than that of Fe/Zn/Ag and 30 times higher than that of Fe/(Zn/Ag) trimetallic system. The reactivity of the Fe/Zn/Ag system was two times higher than that of the Fe/(Zn/Ag) trimetallic system. This observation suggests that the order of metal addition in the trimetallic system has greatly influenced the reactivity of the trimetallic system. Most studies involving the degradation of pollutants using nZVI and nZVI-based bimetallic and trimetallic nanoparticles fitted the degradation on the pseudo-first-order kinetic model [35,42,77,78,79]. However, there are studies showing a second-order fit for degradation of pollutants by nZVI and iron oxide nanoparticles [80,81]. In these studies, the second-order kinetic fit was mainly attributed to the large access of reducing agent, which promotes the corrosion of iron [80]. These results show that the rate of the reaction is proportional to the square of the concentration of the dye [82]. Moreover, Table 3 shows a comparison of the dye removal efficiencies obtained in previous studies to the one obtained in this study using Fe/Ag/Zn trimetallic nanoparticles. In comparison with previous studies, the Fe/Ag/Zn system reported in this work showed a better catalytic activity and behaved similarly to the Fe/Cu/Ag system [36]. Moreover, the Fe/Ag/Zn system was able to remove 100% of the dye within 1 min into the reaction at a lower nanoparticle dosage, pH and dye concentration than in the other studies. Furthermore, despite the lower dye concentration in this study, the results show that the Fe/Ag/Zn system performed better than some previous studies with supported nanoparticles at a relatively lower nanoparticle dose. This, therefore, shows that dye degradation in this study was more rapid and efficient than in the reported previous studies.

However, the reactivity of nZVI-based trimetallic nanoparticles and mechanism has not been clearly stated and remains incomprehensible and controversial [35]. There are previously hypothesized mechanisms of the reaction of trimetallic nanoparticles in the degradation of organic pollutants. The degradation process has been said to either proceed by the adsorption of atomic hydrogen onto the transition metal additive [83] and/or that the transition metal may produce atomic hydrogen that is surface-bound [72,84]. In addition, the surface additives could boost the reactivity by increasing the iron corrosion rate by forming galvanic couples due to the potential difference between nZVI and additives [72].

**Table 3:** Reported dye removal percentages by nZVI, bimetallic and trimetallic nZVI nanoparticles in comparison with the current study.

	NP Used	Dosage (mg/L)	pH	Temperature (°C)	Dye Concentration (mg/L)	Removal %	Reaction Time (min)	References
1	nZVI	150	7.0	Room temperature	MO-40	98	30	[85]
2	B nZVI	500	6.5	30	MO-100	79.5	10	[86]
3	B* nZVI	600	4.1	Room temperature	MO-300	98.5	10	[87]
4	B Fe/Pd	500	6.2	25	MO-200	91.9	20	[25]
5	Fe/Ni	3000	-	28 ± 2	Orange G-150	99.9	10	[88]
6	Fe/Cu/Ag	200	3.0	Room temperature	MO-10	100.0	1	[36]
7	Fe/Ag/Zn	200	3.0	Room temperature	MO-10	100.0	1	Current study

B—Bentonite supported, B*—Biochar supported.

### 3.3. Evaluating the Catalytic Activity of the Trimetallic Systems

The number of active sites of the trimetallic nanoparticles was used to establish the sites on which the reaction occurs [89]. These were equated to the number of moles of the metal ions in a specific quantity of catalyst for a certain reaction [89], 10 mg catalyst loading in this case. The results of the active sites, BET surface area, pore dimensions, *TON* and *TOF* calculations are summarized in Table 4. The number of active sites for all three trimetallic nanoparticles is the same because the ratios of the metals used in each system are the same. However, the *TON* and *TOF* values decreased in the order Fe/Ag/Zn > Fe/Zn/Ag > Fe/(Zn/Ag), consistent with the degradation efficiency and kinetic trends discussed earlier. Although the total number of active sites of the nanoparticles is the same, their catalytic effectiveness varies. This suggests that in the Fe/(Zn/Ag) and Fe/Zn/Ag systems, a portion of the active sites may be inaccessible or not directly involved in the catalytic reaction [90], as inaccessible surface sites typically result in lower catalytic activity. Catalytic active sites are said to be responsible for the reactivity of the catalyst; thus, the inaccessibility of the active sites leads to a decline in catalytic activity [91]. TEM images further support this interpretation, showing that Fe/Ag/Zn nanoparticles are less agglomerated than Fe/Zn/Ag, meaning that more surface sites remain exposed. This agrees with the observation that dispersed nanoparticles possess more accessible active sites than agglomerated ones [92].

Additionally, BET surface area measurements revealed that the Fe/Ag/Zn system exhibits a higher surface area than the other systems, indicating a greater number of available adsorption reaction sites for dye molecules. In addition to surface area, the pore structural parameters revealed that all three materials possess mesoporous features, with average pore diameters in the 1.71–26.62 nm range. The Fe/Ag/Zn nanoparticles show a broader and more favorable pore area distribution compared to Fe/Zn/Ag and Fe/(Zn/Ag). The wider pore area range in Fe/Ag/Zn suggests more effective diffusion pathways and a greater number of accessible adsorption–reaction sites [93]. In contrast, Fe/(Zn/Ag) exhibits the lowest pore volume and pore area, which is consistent with its lower catalytic performance. These textural differences further corroborate the superior accessibility and utilization of the active sites in the Fe/Ag/Zn catalyst. The higher surface area, together with the favorable pore characteristics and reduced agglomeration, aligns well with the superior *TON* and *TOF* values of Fe/Ag/Zn. The *TOF* of the Fe/Ag/Zn catalyst was 0.7461 min^−1^, meaning that 0.7461 moles of dye were converted by one active site per minute, further confirming its superior catalytic activity.

### 3.4. Electrochemical Studies of the Nanoparticles

**Figure 7:**
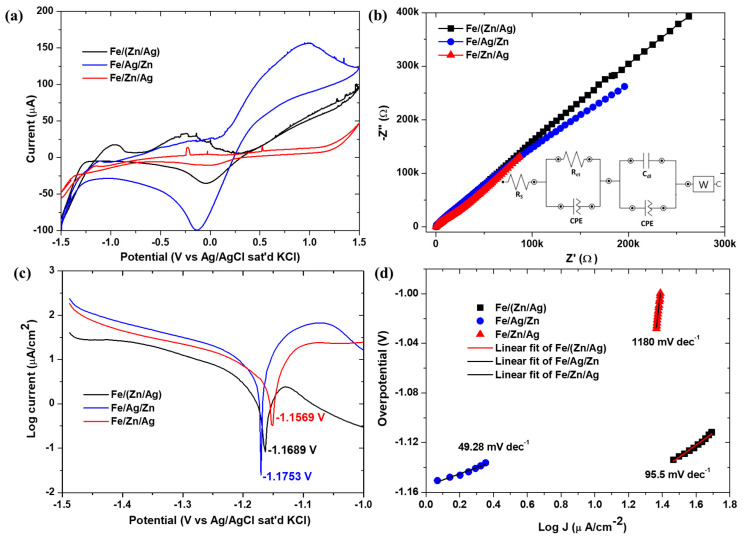
(**a**) Cyclic voltammetry curves. (**b**) Nyquist impedance plots (inset: Randles equivalent circuit). (**c**) Tafel polarization curves of the modified electrodes. (**d**) Corresponding cathodic Tafel slopes in the presence of MO.

The cyclic voltammograms (Figure 7a) of the three modified electrodes exhibit well-defined redox features, revealing clear differences in catalytic activity and electron transfer behavior. Both the position and intensity of the redox peaks vary significantly among the Fe/Ag/Zn, Fe/(Zn/Ag), and Fe/Zn/Ag electrodes. The Fe/Ag/Zn modified electrode shows the highest current response, indicating enhanced electrocatalytic reactivity compared to the other two electrodes. In addition, the overall shape of the CV curves suggests that the capacitive behavior likely includes contributions from Faradaic processes associated with reversible redox activity. This deviates from the more rectangular profile typically expected for purely electric double-layer capacitance, indicating that pseudocapacitive effects may be present [94,95]. However, a rigorous quantification of pseudocapacitive vs. double-layer contributions would require additional analyses (e.g., systematic scan rate dependence and separation of capacitive and diffusion-controlled currents), which were beyond the scope of this study. Therefore, the present discussion is limited to a qualitative interpretation of the CV response. In the Fe/Ag/Zn-modified electrode, a well-defined redox couple is observed, with the anodic peak occurring at approximately +158 μA at around +0.98 V, accompanied by a corresponding cathodic peak during the reverse scan. This behavior is consistent with redox processes involving iron-containing species (e.g., Fe^0^/FeOx/Fe^2+^/Fe^3+^) [96,97], and its high intensity indicates efficient electron transfer and strong catalytic activity. Normalization of the anodic peak yields a geometric current density of 2236 μA cm^−2^ and a mass-normalized current of 3160 μA mg^−1^, highlighting the high intrinsic electroactivity of the Fe/Ag/Zn composite. In contrast, the Fe/(Zn/Ag) electrode exhibits only a modest anodic peak of 32.3 μA, corresponding to 457 μA cm^−2^ and 646 μA mg^−1^, indicating substantially weaker charge transfer behavior. The Fe/Zn/Ag electrode shows no discernible anodic peak within the same potential range, suggesting minimal electroactive involvement. Given the identical catalyst loading and preparation protocols used for all electrodes, these results clearly demonstrate the superior electrochemical performance of the Fe/Ag/Zn electrode. All three electrodes exhibit cathodic current responses as the potential shifts toward more negative values, reflecting reduction reactions. Among them, the Fe/Ag/Zn electrode displays the most pronounced reduction current response, reaching approximately −100 μA at −0.137 V. The Fe/(Zn/Ag) electrode shows moderate cathodic behavior with currents near −80 μA, whereas the Fe/Zn/Ag electrode presents only minimal cathodic activity, indicating limited reactivity in the reduction potential region. The reduction peaks represent the electrochemical reduction reaction involving iron species in different oxidation states [97,98].

Surface electron transfer is generally regarded as an important pathway contributing to contaminant degradation by nZVI [99]. To further evaluate the electron transfer efficiency of the as-synthesized nanoparticles, the Tafel polarization measurements and electrochemical impedance spectroscopy (EIS) were performed. These techniques provide complementary insights into the kinetics of interfacial electron transfer and the overall charge transfer resistance (Rct) of the nanomaterials. The Nyquist plots and the Randles equivalent circuit of the Fe/Ag/Zn, Fe/(Zn/Ag), and Fe/Zn/Ag electrodes are illustrated in Figure 7b. The typical Nyquist plots display a semicircle in the low-frequency region, with its diameter representing the Rct of the modified electrodes. Additionally, a straight line at a 45° angle to the horizontal axis in the low-frequency region indicates a diffusion-limited process characterized by the Warburg element [100]. The Randles circuit showed characteristic elements, solution resistance (Rs), Rct, constant phase element (CPE), double-layer capacitance (Cdl), and Warburg element. The fitted EIS results reveal that the Fe/Ag/Zn electrode exhibits a low Rct of 164 Ω compared with the significantly higher values measured for Fe/(Zn/Ag) (916 Ω) and Fe/Zn/Ag (3.60 kΩ). This substantially reduced Rct indicates that the Fe/Ag/Zn electrode facilitates faster interfacial electron transfer, reflecting its enhanced electrochemical performance. These efficient charge transport characteristics align well with the enhanced catalytic activity observed in the voltammetric measurements. Nevertheless, R_ct_ alone does not constitute direct mechanistic proof of the degradation pathway; rather, it provides supportive evidence that the Fe/Ag/Zn electrode offers a more conductive and electrochemically active interface, which is favorable for electron-mediated degradation reactions.

The CV and EIS measurements (Figure S4, Supplementary Materials) were also recorded in the absence of MO. These control experiments allow us to distinguish the intrinsic redox and charge transfer behavior of the modified electrodes from MO-related effects, and to confirm that the Fe/Ag/Zn electrode maintains its superior electrochemical performance under degradation-relevant conditions. In the absence of MO, the Fe/Ag/Zn electrode still displays an enhanced current response in the potential region associated with iron redox transitions, together with a lower charge transfer resistance (181 kΩ) compared with Fe/(Zn/Ag) (320 kΩ) and Fe/Zn/Ag (953 kΩ). Comparison of the spectra shows that the presence of MO leads to an increase in current response and a further decrease in charge transfer resistance that are most pronounced for the Fe/Ag/Zn electrode, whereas the changes for Fe/(Zn/Ag) and Fe/Zn/Ag are considerably smaller. These observations indicate that MO participates in interfacial electron transfer processes and that the Fe/Ag/Zn electrode maintains particularly efficient charge transfer in the presence of the MO.

The Tafel polarization analysis (Figure 7c) and corresponding Tafel slopes (Figure 7d) demonstrate that the Fe/Ag/Zn electrode exhibits enhanced electron transfer capability. This is evidenced by its significantly more negative free corrosion potential (−1.174 V) and its low Tafel slope (49.28 mV dec^−1^), both of which are lower than those of the Fe/(Zn/Ag) and Fe/Zn/Ag electrodes. A more negative corrosion potential indicates a greater tendency for the material to release electrons, while a lower Tafel slope reflects faster charge transfer kinetics. Together, these results show that the Fe/Ag/Zn system is more electrochemically active, more prone to corrosion, and capable of supporting a higher electron-migration rate than the other electrode configurations [100,101].

### 3.5. Effect of Reaction Conditions on Catalyst Performance

The effect of operational parameters on the degradation of MO dye in aqueous solution using the Fe/Ag/Zn trimetallic nanoparticles was investigated. The Fe/Ag/Zn trimetallic system was used due to its excellent reactivity for MO degradation compared with the other trimetallic systems prepared in this study. The catalytic decolorization and degradation of MO dye using the trimetallic nanoparticles were carried out using the second-order kinetic model.

#### 3.5.1. Effect of Initial Solution pH

**Figure 8:**
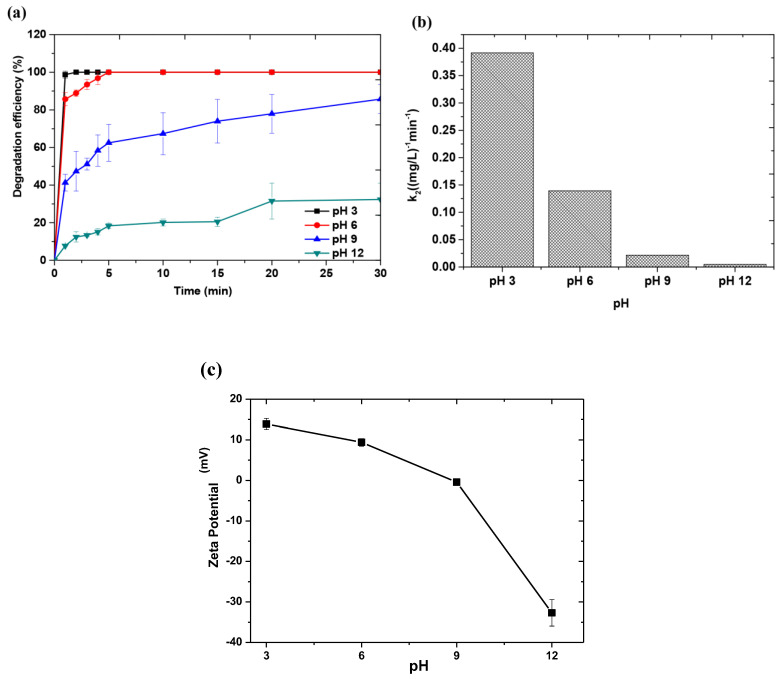
The degradation efficiencies of 10 mg/L methyl orange dye using 10 mg Fe/Ag/Zn 5:0.1:5 nanoparticles at different pH values (**a**), the second-order rate constants at different pH values (**b**) and zeta potential of Fe/Ag/Zn 5:0.1:5 nanoparticles measured across different pH values (**c**).

The pH of the solution plays a significant role in the degradation process of different organic pollutants and thus it was of interest to study its effect on MO degradation using Fe/Ag/Zn trimetallic nanoparticles [102,103,104]. The effect of pH on the degradation of MO was studied in the pH range 3–12 and the degradation results are shown in Figure 8a. Solution pH was adjusted prior to degradation tests. Figure 8a shows that the degradation efficiency of MO decreases with an increase in solution pH. At lower pH values (pH 3 and 6), the degradation efficiency of MO occurs rapidly and reaches 100% within 5 min. When the pH of the solution was increased to 9 and 12, the degradation efficiencies of MO drastically decreased. The enhancement in degradation efficiency of MO at low pH could be attributed to the high concentration of hydrogen ions which are actively involved in nZVI reductive reaction [105]. At high pH values, the decline in degradation efficiency is primarily due to the development of ferrous/ferric hydroxide complexes made from Fe (III), Fe (II) and OH^−^ ions, resulting in the deactivation of the nZVI catalyst [106]. The second-order degradation rate constants are shown in Figure 8b. It can be observed that the rate constants decrease with an increase in pH values, 0.3917, 0.1393, 0.0217 and 0.0047 (mg/L)^−1^ min^−1^ at pH 3, 6, 9 and 12, respectively. The second-order rate constant at the pH of 3 was 83 times higher than that at pH 12 and 18 times higher than that at pH 9. Thus, the highest reaction rate of degradation is obtained at lower pH and these results are similar to what other researchers have previously reported [87,107].

Zeta potential measurements were also performed to assess the surface charge behavior of the Fe/Ag/Zn nanoparticles across the tested pH range (Figure 8c). The zeta potential decreased progressively with increasing pH, indicating that the particles are strongly positively charged under acidic conditions and become less positive at neutral and alkaline pH. This behavior is consistent with previously reported trends for nZVI and Fe-based nanoparticles, where protonation at low pH promotes a higher positive surface charge, while deprotonation reduces surface charge as pH increases [108,109]. The strongly positive charge at pH 3 enhances electrostatic attraction toward anionic MO molecules, complementing the observed rapid degradation under acidic conditions. As the surface charge decreases at higher pH values, this interaction weakens, contributing to the reduced degradation efficiencies observed in alkaline conditions. Therefore, it can be inferred that acidic conditions are best suited for the degradation of MO by Fe/Ag/Zn trimetallic nanoparticles.

#### 3.5.2. Effect of Initial Methyl Orange Dye Concentration

**Figure 9:**
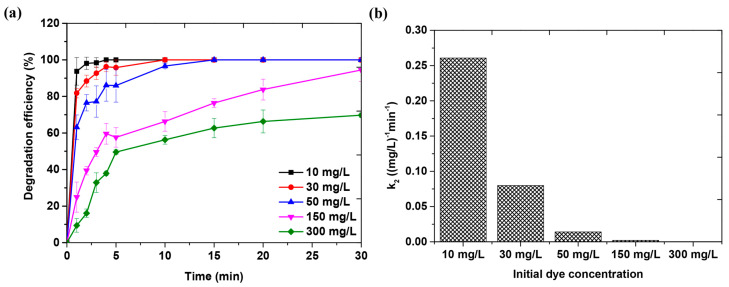
The degradation efficiencies of 10 mg/L methyl orange dye using 10 mg Fe/Ag/Zn 5:0.1:5 nanoparticles at different initial dye concentrations (**a**) and the apparent rate constants at different methyl orange dye concentrations (**b**).

The influence of initial MO dye concentration of the degradation efficiency by Fe/Ag/Zn system is shown in Figure 9a. The plot shows that the degradation efficiency of MO decreases with an increase in initial MO concentration. However, the lower initial concentrations (10, 30 and 50 mg/L) reached 100% degradation within 15 min into the reaction, though degradation occurs more rapidly at the initial MO concentration of 10 mg/L where 100% apparent degradation is obtained within 2 min. When the initial concentration was increased to 150 and 300 mg/L, the degradation efficiency decreased. Furthermore, the results for the rate of reaction of MO degradation at different initial concentration are summarized in Figure 9b on the plot of k_2_ versus initial dye concentration. The second-order rate constants were 2.6069, 0.0799, 0.0141, 0.0020 and 2.296 × 10^−4^ (mg/L)^−1^ min^−1^ for 10, 30, 50, 150 and 300 mg/L, respectively. The second-order rate constants of MO degradation by the Fe/Ag/Zn nanoparticles decreased drastically with an increase in the initial dye concentration. The rate constant at the initial dye concentration of 10 mg/L was 33 times higher than that at 25 mg/L, 185 times higher than that at 50 mg/L, 1291 times higher than that at 150 mg/L and 11,351 times higher than that at 300 mg/L. This shows that at lower dye concentrations, the rate of the reaction is extremely fast. Similar observations of decreasing degradation efficiencies with increasing initial pollutant concentration have been previously reported [110,111]. This trend can be attributed to the rapid occupation of all the trimetallic nanoparticles active sites by MO molecules at high dye concentration [111]. Furthermore, the particles’ adsorption capacity is limited and the increase in MO concentration leads to competitive adsorption among MO molecules whereby the adsorbed molecules will impede the adsorption and reduction in other molecules in the solution [41,110].

#### 3.5.3. Effect of Initial Nanoparticle Dosage

**Figure 10:**
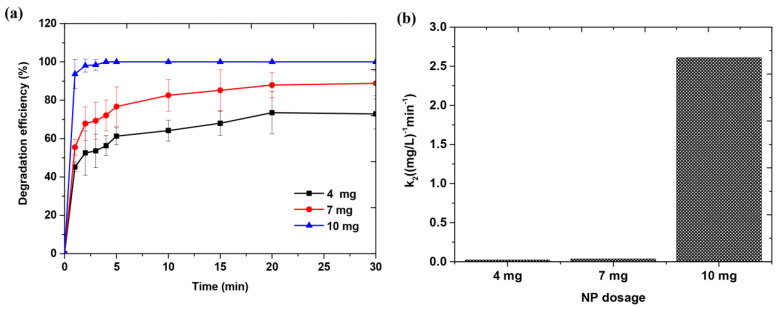
The degradation efficiencies of 10 mg/L methyl orange dye using Fe/Ag/Zn 5:0.1:5 nanoparticles at different initial nanoparticle dosages (**a**) and the apparent rate constants at different initial nanoparticle dosages (**b**).

The effect of Fe/Ag/Zn trimetallic nanoparticle loading on the degradation of MO was studied with three different catalyst loadings (4, 7 and 10 mg). As depicted in Figure 10a, the degradation efficiency of MO increases with increasing nanoparticle dosage. The lowest loadings of 4 and 7 mg do not reach 100% degradation within the set reaction time of 30 min. On the contrary, the 10 mg nanoparticle loading reached 100% apparent degradation within a 2 min reaction time. Moreover, the second-order rate constants increase with an increase in nanoparticle dosage as shown in Figure 10b. The degradation rate constants are 0.0212, 0.0343 and 2.6069 mg/L^−1^min^−1^ for 4, 7 and 10 mg nanoparticle loadings, respectively. The k_2_ at the initial nanoparticle dosage of 10 mg was 76 times higher than that at 7 mg and 123 times higher than that at 4 mg dosage. These observations may be ascribed to the fact that at a high nanoparticle dosage there are more particles that can provide more active surface sites for collision with MO dye molecules to achieve greater dye removal efficiency [105,112]. This was an expected observation as the same was found to be the case in previous studies involving degradation of organic pollutants by nZVI and nZVI-based particles [86,105].

### 3.6. Methyl Orange Degradation Products and Pathway

**Figure 11:**
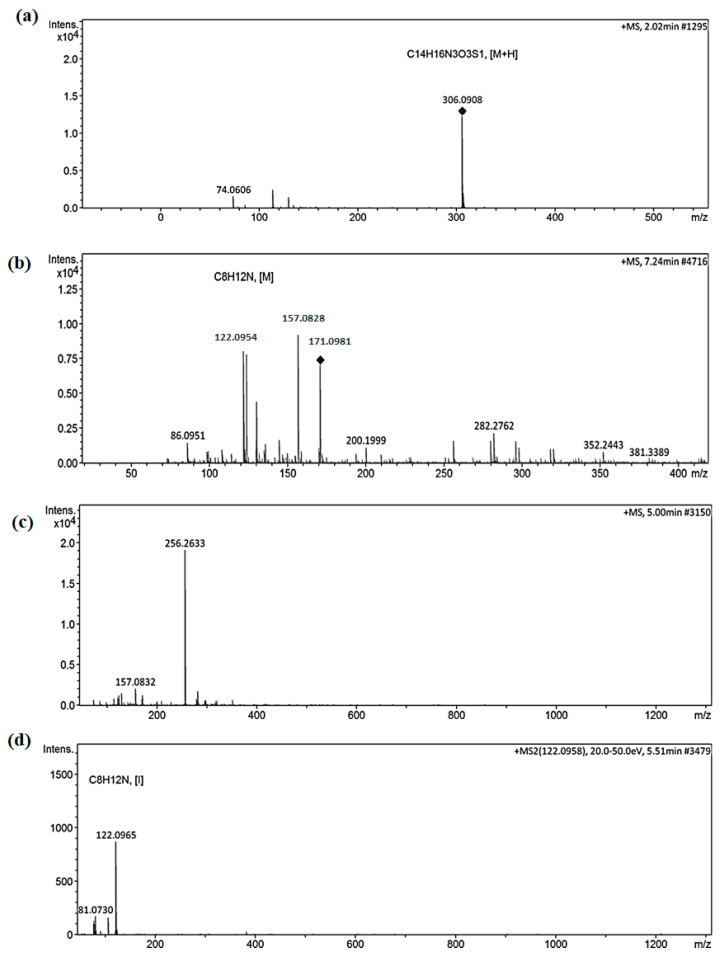
Total ion chromatograms of methyl orange degradation at (**a**) 0 min, (**b**) 1 min, (**c**) 30 min at retention time of 5.51 min and (**d**) 30 min at retention time of 5.00 min.

The degradation pathway and products from MO degradation using Fe/Ag/Zn trimetallic nanoparticles were determined using LC-MS analysis. The total mass chromatograms of MO and its degradation products at 0, 1 and 30 min of degradation are shown in Figure 11. According to the results obtained by UV-vis analysis, the degradation of MO occurs within 1 min. Similarly, from the LC-MS results, it can be seen that the peak for MO eluted at 2.02 min retention time on the chromatogram and completely disappears within 1 min (Figure S5, Supplementary Materials). The mass spectrogram in Figure 11a at 0 min has an identified peak at m/z value of 306 corresponding to MO dye before degradation. Figure 11b shows the mass spectrogram at 1 min which shows peaks at m/z values of 122, 157 and 171 which were identified as N,N dimethyl benzeneamine, benzenesulfonic acid and sulfanilic acid, respectively. These were due to the symmetrical cleavage of the azo bond on the MO structure. In this case, the degradation occurred rapidly compared to some previous studies involving the degradation of MO dye [113,114,115]. Generally, before the appearance of peaks at m/z 157 and 121, the total mass spectrogram from MO degradation shows peaks at m/z 172 and 136 which are directly due to the cleavage of the azo bond [114,115].

**Figure 12:**
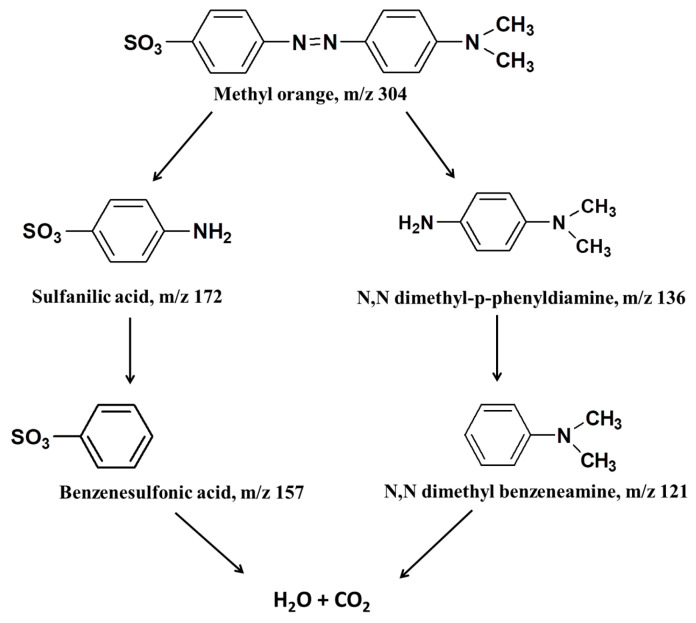
Possible degradation pathway of methyl orange using Fe/Ag/Zn 5:0.1:5 nanoparticles.

Moreover, Figure 11c and Figure 11d show the total mass chromatograms at 30 min at retention times of 5.51 and 5.00 min, respectively. Figure 11c has a peak at m/z 157 and Figure 11d has a peak at m/z 122 which were both identified above as products from MO degradation. However, these products have both reduced in intensity, proving their disappearance as degradation duration increases. Thus, with an increase in reaction time and introduction of other parameters favoring mineralization, there is a possibility of formation of CO_2_ and H_2_O as the products from MO degradation are more prone to mineralization than MO itself [116,117,118]. The peak at m/z 256 on Figure 11c could not be identified as it does not correspond to masses of the products from MO degradation or the nanoparticles used in the study. Thus, it could potentially be due to impurity ions that can enter the mass analyzer through the column, the sample itself or mobile phase solvents providing peaks with high intensities on the total ion chromatogram [119]. Using the above information, a degradation pathway was identified as shown in Figure 12. The degradation of methyl orange is proposed to proceed via initial reductive cleavage of the azo bond, leading to the formation of aromatic amine intermediates such as sulfanilic acid and N,N-dimethyl-p-phenylenediamine. These intermediates may undergo further oxidation and transformation through subsequent redox reactions in the system. While continued breakdown of these products could ultimately result in mineralization, this pathway cannot be conclusively confirmed within the scope of the present study.

### 3.7. Probable Methyl Orange Degradation Mechanism by Fe/Ag/Zn Trimetallic Nanoparticles

The mechanism for degradation or removal of organic pollutants by trimetallic nanoparticles has not been identified and is deemed controversial [76]. However, herein, an explanation for a hypothetical possible degradation mechanism for the MO dye by trimetallic nanoparticles was attempted. Studies have shown that the degradation of dyes by nZVI is a redox reaction and can occur in two ways: oxidation in the presence of dissolved oxygen and reduction in anaerobic conditions [120,121]. The Fe/Ag/Zn system contains two reducing agents (Fe^0^ and Zn^0^), which are both strong reducing agents generating reactive electrons that facilitate dye reduction [121,122]. Since the experiments were performed in an open-batch system, before the invasion of oxygen into the mixture, the following reactions can take place:

(12) $${Fe}^{0}+2H_{2}O\to {Fe}^{2+}+H_{2}+2OH^{-}$$

(13) $${Fe}^{0}+2H^{+}\to {Fe}^{2+}+H_{2}$$

(14) $${Zn}^{0}+2H_{2}O\to {Zn}^{2+}+H_{2}+2OH^{-}$$

Equations (12)–(14) show the corrosion of Fe^0^ and Zn^0^ in the presence of water and H^+^. In this case there are various potential reducing agents for the MO dye in the system (Fe^0^, Zn^0^, Fe^2+^, Zn^2+^ and H_2_) [121]. In the open-batch system, dissolved O_2_ can be reduced to superoxide radicals (O_2_•^−^) (Equation (15)), which may further react with protons to form hydrogen peroxide (H_2_O_2_).

(15) $$O_{2}+e^{-}\to O_{2}{•}^{-}$$

Therefore, as the reaction continues in the presence of dissolved oxygen, the corrosion of Fe^0^ can happen as follows:

(16) $${Fe}^{0}+O_{2}+2H^{+}\to {Fe}^{2+}+H_{2}O_{2}$$

(17) $${Fe}^{0}+H_{2}O_{2}+2H^{+}\to {Fe}^{2+}+2H_{2}O$$

(18) $${Fe}^{2+}+H_{2}O_{2}\to {Fe}^{3+}+•OH+OH^{-}$$

Equations (16)–(18) describe the oxidation of Fe^0^ in the presence of dissolved oxygen, leading to the in situ formation of H_2_O_2_. The generated H_2_O_2_ subsequently reacts with Fe^2+^ to yield hydroxyl radicals (•OH) through a Fenton-like process (Equation (18)) [123]. These highly reactive species serve as potent oxidizing agents, attacking the MO dye molecules and their degradation intermediates. Lastly, the purpose of Ag nanoparticles could be to enhance interfacial charge transfer within the Fe/Ag/Zn system by acting as an efficient electron mediator between Fe and Zn domains. This promotes faster redox cycling and inhibits charge recombination, thereby sustaining a high density of active electrons in the system for pollutant degradation [28,75,124]. Collectively, these radicals, together with the reducing activity of Fe^0^/Zn^0^ and the electron-mediating role of Ag, drive the rapid decolorization and eventual mineralization of MO.

**Figure 13:**
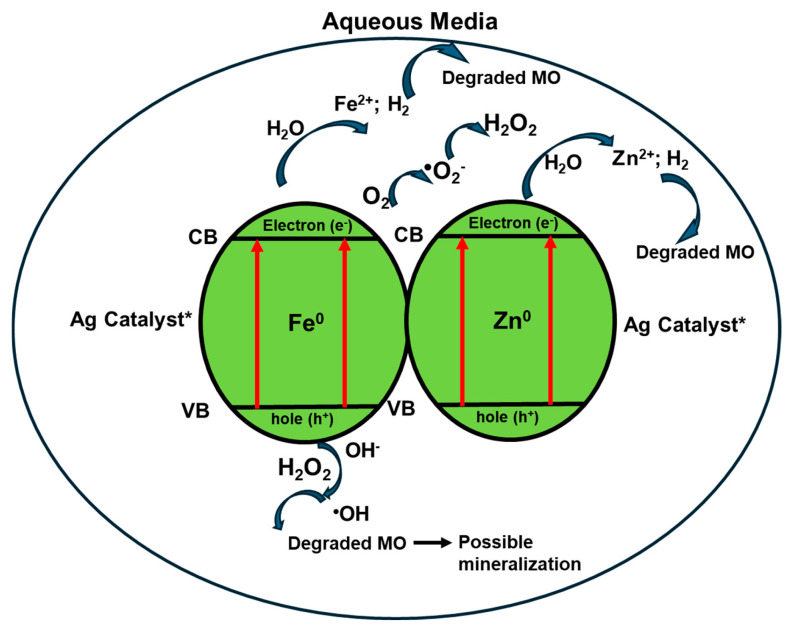
Summary of the probable mechanism of MO degradation by Fe/Ag/Zn 5:0.1:5 trimetallic nanoparticles in aqueous media. * = Ag catalyst reduces the potential of charge recombination and promotes redox recycling.

In summary, the degradation of MO likely begins with the cleavage of the azo bond, initiated by reduction through active reducing agents (see Figure 13). This is followed by the formation of aromatic amines via protonation from water, which serves as a proton donor, assisted by electrons released during corrosion [125,126]. Subsequently, dissolved oxygen in the system promotes the generation of •OH, which can further oxidize and mineralize the degradation intermediates. The mineralization process is proposed based on the observed intermediates; however, further studies are required to confirm this step conclusively. This study demonstrated higher degradation efficiency at lower pH values, which can be attributed to the enhanced dissolution of Fe^0^ and the subsequent release of Fe^2+^ ions. The increased Fe^2+^ concentration promotes Fenton-like oxidation, generating a higher concentration of •OH essential for effective mineralization [127]. Overall, the synergistic interaction among Fe, Ag and Zn enhances the formation of reactive species, leading to rapid and efficient degradation of the dye. The catalytic degradation of MO in this system likely proceeds through a combined redox–Fenton-like pathway.

In situ XPS or XAS studies at the Fe K-edge, Zn K-edge, and Ag L_3_-edge would provide direct evidence of valence-state transitions (Fe^0^ → Fe^2+^/Fe^3+^, Zn^0^ → Zn^2+^) and the electron enrichment of Ag during catalysis. Although not performed in this study, these analyses are planned for future work to further validate the proposed redox mechanism and to gain deeper insight into the dynamic charge redistribution among the three metals. Overall, the Fe/Ag/Zn trimetallic nanoparticles exhibited excellent catalytic activity under mild reaction conditions. Furthermore, reusability, post-reaction structural stability, and metal leaching will be systematically investigated in future studies to evaluate the long-term performance and environmental relevance of the Fe/Ag/Zn system. Their simple, cost-effective synthesis and scalability, combined with their high degradation efficiency, suggest that this system holds strong potential for practical application in industrial wastewater treatment processes.

## 4. Conclusions

In this study, three trimetallic nanoparticles (Fe/Ag/Zn, Fe/Zn/Ag and Fe/(Zn/Ag)) with different metal addition sequences were successfully synthesized, characterized and tested. The TEM, XRD, SEM and XPS analyses confirmed the successful synthesis of these nanoparticles with TEM and EDS mapping images showing chain-like structure of the particles due to magnetic interactions between them. The catalytic activity of the Fe/Ag/Zn was found to be higher than those of the other trimetallic systems with the *TOF* value of 0.7461 min^−1^. This showed that the performance of the trimetallic nanoparticles was affected by the sequence of metal addition onto the surface of nZVI. The degradation efficiency of MO by the Fe/Ag/Zn nanoparticles and the reactivity of the nanoparticles were shown to be greatly affected by the pH, initial MO dye concentration and nanoparticle dosage. The k_2_ at pH 3 was 83 times higher than that at pH 12 and 18 times higher than that at pH 9. At the initial nanoparticle dosage of 10 mg the k_2_ was 2.6069 (mg/L)^−1^min^−1^ which was 76 times higher than that at 7 mg and 123 times higher than that at 4 mg dosage. The k_2_ at the initial dye concentration of 10 mg/L was multiple times higher than the ones at higher initial dyes concentrations. In general, the process was proven to be highly acid-driven. The results of LC-MS analysis show that apparent MO degradation occurred within 1 min and products formed were more prone to mineralization than the MO dye. Thus, the Fe/Ag/Zn trimetallic nanoparticles demonstrated excellent short-term catalytic performance for the degradation of textile wastewater contaminated with dyes such as MO, owing to synergistic interactions among the metallic components that enhance electron transfer and promote the rapid generation of reactive species.

## Figures and Tables

**Table 1:** Atomic compositions of the trimetallic nanoparticles calculated from XPS spectra.

Sample	Element	Peak Binding Energy (eV)	Atomic %
Fe/Zn/Ag	C 1s	286.0	19.7
	O 1s	533.0	48.7
	Fe 2p	712.4	2.5
	Zn 2p	1022.9	14.0
	Ag 3d	368.8	0.1
Fe/(Zn/Ag)	C 1s	286.0	25.6
	O 1s	532.9	45.4
	Fe 2p	713.4	6.8
	Zn 2p	1022.8	10.9
Fe/Ag/Zn	C 1s	285.9	43.2
	O 1s	533.4	33.3
	Fe 2p	712.9	2.3
	Zn 2p	1023.0	9.0

**Table 2:** R^2^ values of the zeroth-, first- and second-order kinetic data of the trimetallic nanoparticles.

	R^2^ Values		
	Zeroth-Order	First-Order	Second-Order
Fe/(Zn/Ag)	0.6363	0.7229	0.8052
Fe/Zn/Ag	0.7655	0.8578	0.9506
Fe/Ag/Zn	0.1662	0.6537	0.9107

**Table 4:** BET surface area, textural parameters, number of active sites, *TON* and *TOF* values of the trimetallic nanoparticles, with *TON* and *TOF* calculated at 5 min reaction time.

	Degradation Efficiency (%)	Number of Active Sites (moles)	*TON*	*TOF* (min^−1^)	BET Surface Area (m^2^/g)	Average Pore Diameter * (nm)	Pore Volume * (cm^3^/g)	Pore Area * (m^2^/g)
Fe/(Zn/Ag)	42.21	4.09 × 10^−5^	1.5669	0.3134	30.4406	1.71–24.67	0.03–0.07	5.98–28.81
Fe/Zn/Ag	46.73	4.09 × 10^−5^	1.7534	0.3507	37.0288	1.78–25.07	0.06–0.13	10.37–42.25
Fe/Ag/Zn	100.00	4.09 × 10^−5^	3.7307	0.7461	78.3437	1.73–26.62	0.16–0.27	17.85–56.93

* Reported values reflect the measured ranges for each corresponding textural property.

## Data Availability

The original contributions presented in this study are included in the article/Supplementary Material. Further inquiries can be directed to the corresponding author.

## Supplementary Materials

The following supporting information can be downloaded at https://www.mdpi.com/article/10.3390/nano16010060/s1.
