# Supplementary material for: Catalytic Degradation of Methyl Orange Using Fe/Ag/Zn Trimetallic Nanoparticles
Source: Nanomaterials (Basel). 2025 Dec 31;16(1):60. doi: 10.3390/nano16010060 (PMC12787407; doi:10.3390/nano16010060)
Supplement: Supplementary file 1 [file nanomaterials-16-00060-s001.zip › nanomaterials-3983142-supplementary.pdf]

Supplementary Material: Catalytic Degradation of Methyl Orange Using Fe/Ag/Zn Trimetallic Nanoparticles

Masaku Kgatle[1,2*], Keneiloe Khoabane[1], Ntsoaki Mphuthi[1], Gebhu Ndlovu[1] and Nosipho Moloto[2]

[1]*DSTI/Mintek Nanotechnology Innovation Centre, Advanced Materials Division, Mintek, Private Bag X3015, Randburg 2125, South Africa.*

[2]*Molecular Sciences Institute, School of Chemistry, University of the Witwatersrand, P/Bag 3, WITS 2050, Johannesburg, South Africa.*

*Corresponding author: masakuk@mintek.co.za

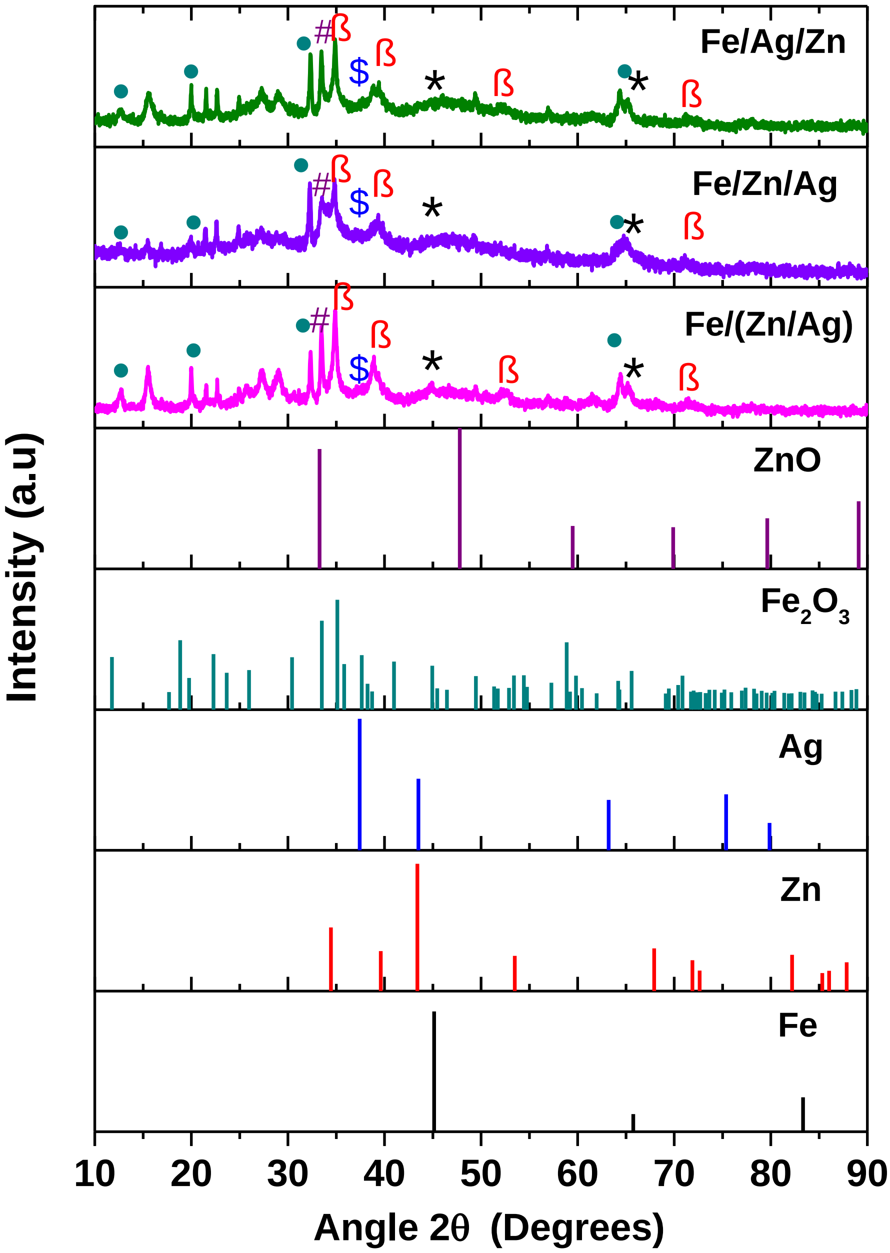

Figure S1: X-ray diffraction (XRD) patterns of the trimetallic nanoparticles and standard XRD plots of Fe, Zn, ZnO and Fe_2O_3. (Peaks of Fe^0: *, peaks of Fe_2O_3: ●, peaks of Ag: $, peaks of Zn: β and peaks of ZnO: #).

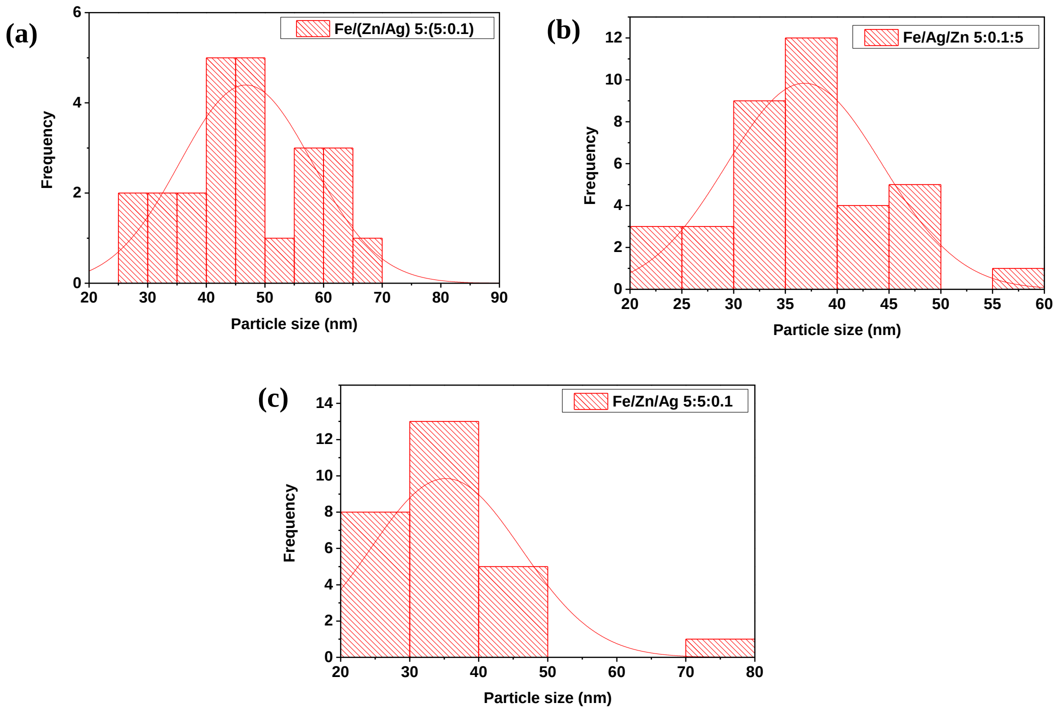

Figure S2: Particle size histograms of trimetallic Fe/(Zn/Ag) 5:(5:0.1) (a), Fe/Ag/Zn 5:0.1:5 (b) and Fe/Zn/Ag 5:5:0.1 (c).

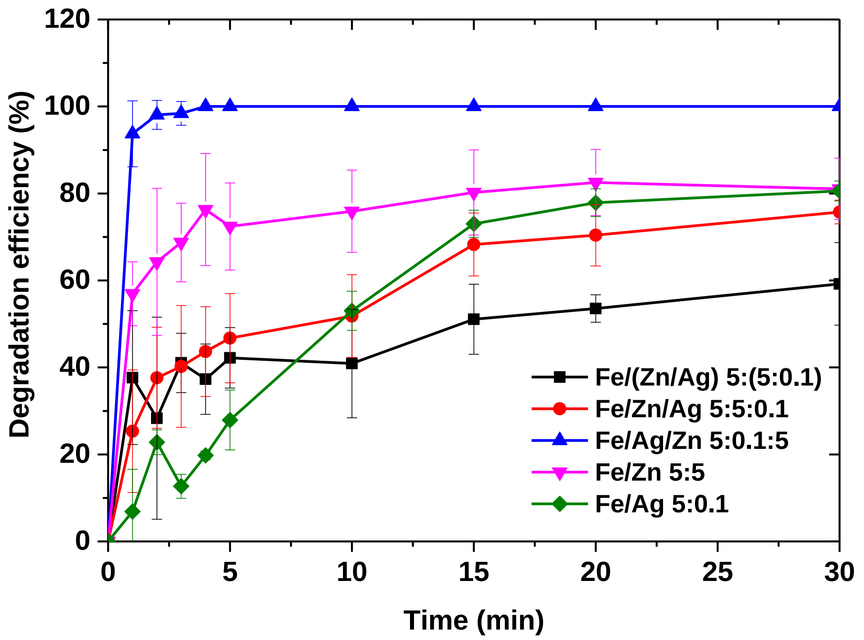

Figure S3: Degradation efficiencies of 10 mg/L methyl orange dye using 10 mg of the trimetallic nanoparticles (Fe/Zn/Ag 5:5:0.1, Fe/Ag/Zn 5:0.1:5 and Fe/(Zn/Ag) 5:(5:0.1)).

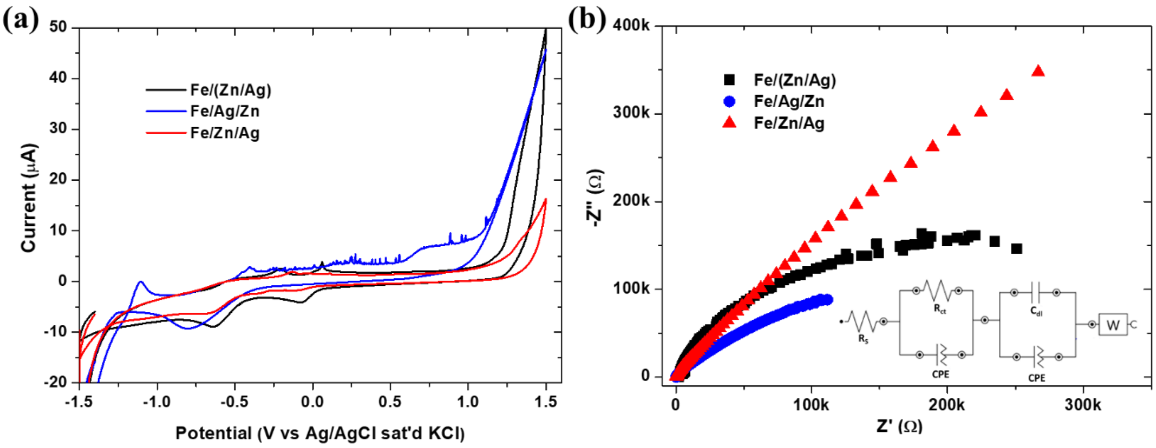

Figure S4: (a) Cyclic voltammetry curves, (b) Nyquist impedance plots (inset: Randles equivalent circuit) in the absence of MO.

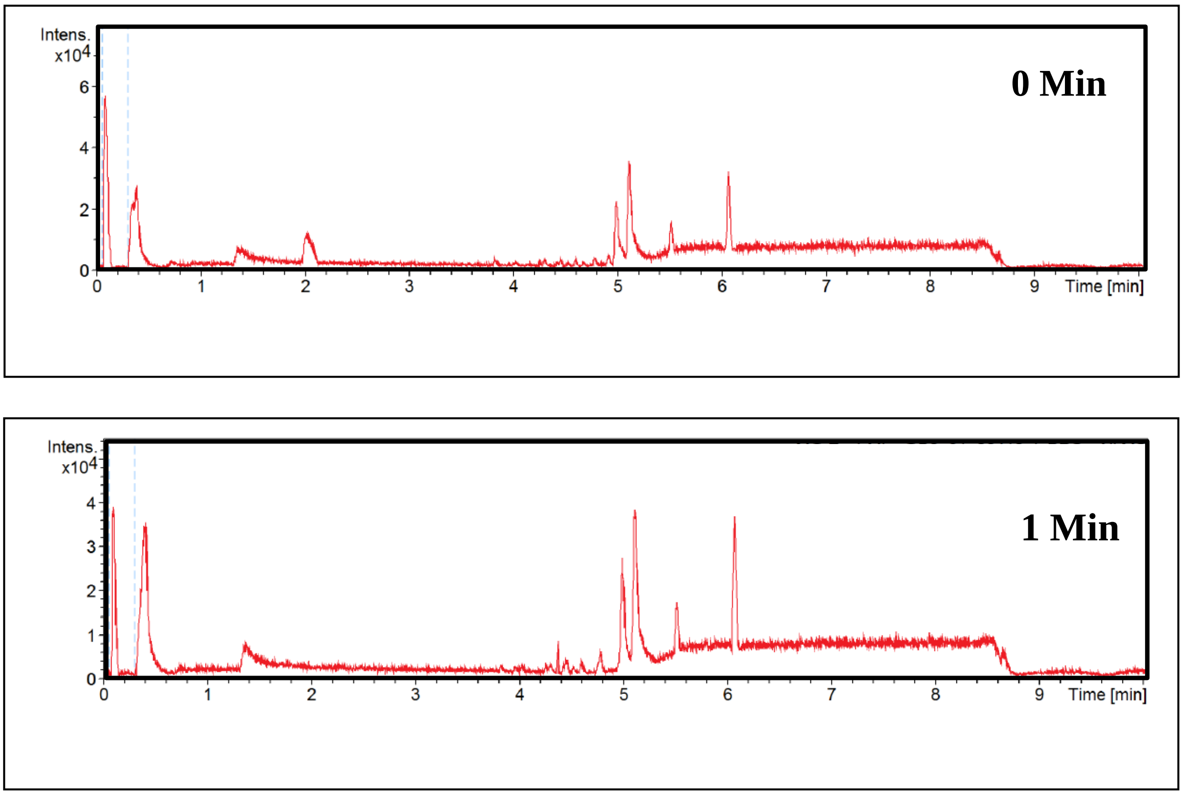

Figure S5: Mass chromatogram of (a) Methyl orange before degradation at 0 minutes and (b) Methyl orange after 1 minute degradation by Fe/Ag/Zn 5:0.1:5 nanoparticles.
